# Entorhinal cortex layer III *Adgrl2* expression controls topographical circuit connectivity required for sequence learning

**DOI:** 10.1038/s41398-025-03490-5

**Published:** 2025-08-08

**Authors:** Jordan D. Donohue, Crisylle Blanton, Anna Chen, Amna Ahmad, Elizabeth D. Liu, Lisette Saab, Rajbir Kaur, Woojin Yang, Garret R. Anderson

**Affiliations:** 1 Department of Molecular, Cell, and Systems Biology; University of California - Riverside, Riverside, CA 92521 USA; 2 Neuroscience Graduate Program, University of California - Riverside, Riverside, CA 92521 USA

**Keywords:** Molecular neuroscience, Genomics, Learning and memory

## Abstract

The entorhinal cortex and hippocampus are interconnected brain regions required for episodic learning and memory. For this functional encoding, correct assembly of specific synaptic connections across this circuit is critical during development. To guide the connection specificity between neurons, a multitude of circuit building molecular components are required, including the latrophilin family of adhesion G protein-coupled receptors (Lphn1-3; gene symbols *Adgrl1-3*). Within this genetic family, *Adgrl2* exhibits a unique topographical and cell-type specific expression patterning in the entorhinal cortex and hippocampus that mirrors connectivity. To investigate the role of *Adgrl2* in a cell-type specific fashion for this circuit, we here created a transgenic mouse (*Adgrl2*^fl/fl^;pOxr1-Cre) with targeted and selective *Adgrl2* deletion in medial entorhinal cortex layer III neurons (MECIII). Using these mice, we find two major input/output circuitry pathways to be topographically shifted with *Adgrl2* deletion in MECIII neurons. These neural connectivity impacts include MECIII axon projections to contralateral MEC layer I, and presubiculum axons to ipsilateral MEC layer III. To test the behavioral consequences of these circuitry alterations, we investigated varying entorhinal cortex dependent behaviors, revealing selective deficits in spatial-temporal sequence learning. Taken together, this study demonstrates that *Adgrl2* expression in MECIII neurons is necessary for the accurate assembly of MEC topographical circuits that support episodic learning.

## Introduction

Episodic learning and memory are dependent on highly specific patterning of connections established during development between pre- and postsynaptic neurons. The visual component of episodic learning for example, requires axonal projections to form between topographically distinct patterns within retina and thalamocortical circuits to encode spatial relationships [1–3]. Likewise, the connections between the entorhinal cortex and hippocampal regions that are required for episodic learning and memory, are topographically organized and cell-type specific [4–6]. These circuits involve spatially separated interconnected brain regions, which require axons to navigate long distances during development to reach their proper target. This developmental process is controlled at multiple stages, starting with axon guidance, followed by synaptic targeting to specific cell-types, before advancing into pre- to postsynaptic synaptic establishment and specification. The development of connectivity patterns in these circuits has been shown to be controlled by molecular gradients that include protein complexes involving the latrophilin gene family of adhesion G protein-coupled receptors (Lphn1-3; gene symbols *Adgrl1-3*) [7–9]. Implicated as essential molecules in a variety of neural developmental processes, latrophilins participate in circuit development that include cell migration, axon targeting, synaptic recognition, and synaptic development [8, 10–12]. To do so, latrophilins form large extracellular macromolecular complexes either directly or indirectly with a growing number of cell-adhesion protein binding partners that include Teneurins (*Ten1-4*) [13, 14], Fibronectin leucine-rich transmembrane proteins (*FLRT1-3*) [15], Uncoordinated-5 (*UNC5a-d*) [16, 17], and Neurexins [18]. Working in concert, these distinct genetic components participate in context dependent extracellular interactions that have multiple effects during development. Amongst these, Teneurin-3 (*Ten3*) and *Adgrl2* exhibit a spatially adjacent and repulsive relationship with one another to control circuit patterning. In hippocampal circuits, *Adgrl2* and *Ten3* expression is topographically distinct for CA1 and subiculum and is required for the connectivity organization between these two regions [8, 19]. In this circuit, *Adgrl2* + CA1 neurons connect with *Adgrl2*+ subiculum neurons, and likewise *Ten3* + CA1 neurons connect with *Ten3*+ subiculum neurons via a reciprocal repulsion mechanism [8]. Similar *Adgrl2* expression and connectivity patterns appear to apply in parahippocampal circuits as well, where we found topographical *Adgrl2* expression for interconnected regions of the presubiculum (PrS), parasubiculum (PaS) and medial entorhinal cortex (MEC) [9]. In the MEC, *Adgrl2* expression is highly enriched for layer II and III pyramidal neurons, and the removal of which leads to selective disruption in PrS to MEC connectivity [9].

To dissect out the cell-type specific function of *Adgrl2* in MEC circuit development, in this study we investigate the role of *Adgrl2* in a select neuron population, MEC layer III pyramidal neurons (MECIII). To accomplish this, we combined *Adgrl2* conditional knock-out mice (*Adgrl2*^fl/fl^) [12] with a transgenic mouse line expressing Cre-recombinase specifically in MECIII neurons under the control of the Oxr1 promoter (pOxr1-Cre) [20]. The resulting transgenic mouse line leads to selective deletion of *Adgrl2* in MECIII neurons of the medial entorhinal cortex (*Adgrl2*^fl/fl^;pOxr1-Cre). With this genetic manipulation, we find that MECIII *Adgrl2* expression is required for controlling the topographical organization of select axon populations arising from the ipsilateral presubiculum and contralateral MECIII neurons. To test the behavioral function of this circuit reorganization, we performed a series of functional tests directed at assessing entorhinal cortex dependent episodic learning and memory. Here we find that spatial learning and memory behaviors are selectively impacted with this genetic manipulation, finding an impairment in the rate of learning of the water t-maze spatial sequence task. Together, this shows MECIII *Adgrl2* expression is important for the connectivity of select MEC inputs, which controls discrete aspects of episodic encoding.

## Materials and methods

### Animals

*Adgrl2*^fl/fl^, *Adgrl2*-mVenus^fl/fl^, pOxr-Cre mice (C57BL/6N-Tg(Oxr-cre)C14Stl/J), Ai14 (B6.Cg-Gt(ROSA)26Sor tm14(CAG-tdTomato)Hze/J) used in this study were described previously [12, 20, 21]. The original mouse lines used for generation of the genotypes described in this study are available through Jackson Laboratory Mouse Repository for distribution. Mice were weaned at 21 days of age and housed in groups of 2 to 5 on a 12-h light/dark cycle with food and water ad libitum in the University of California, Riverside Animal Housing Facility. Male and female mice were used for all experiments. No obvious differences were noted due to sex. Sample sizes were chosen based on our preliminary studies.

### Ethics approval

All procedures involving laboratory animals conformed to National Institutes of Health Guidelines for the Care and Use of Laboratory Mice and were approved by the University of California, Riverside Administrative Panel on Laboratory Animal Care (07/2023), and Administrative Panel of Biosafety (BUA-R208).

### Stereotaxic viral targeting

Adult mice (P35) surgeries were performed with isoflurane anesthesia. Neonatal mice (P3) surgeries were performed with 5-min ice anesthesia. Using a dual manipulator stereotaxic frame (Kopf), stereotaxic coordinates used were: Anterior/Posterior (AP) in relation to Lambda; Medial/Lateral (ML) in relation to midline; and Dorsal-Ventral (DV) in relation to dorsal brain surface. Coordinates used: Adult Proximal MEC (AP + 0.6 mm, ML: +3.2 mm, DV − 2.2 mm); Adult Distal MEC targeting (AP + 0.8 mm, ML + 3.8 mm, DV − 2.4 mm); Adult Proximal PrS targeting (AP + 0.15 mm, ML + 2.4 mm, DV − 1.8 mm); Neonatal P3 MEC targeting (AP + 0.2 mm; ML + 2.1 mm; and DV − 2.0 mm). Concentrated adeno-associated viruses (AAVs) were injected (0.2 – 0.25 µl) with a glass micropipette tip using a controlled rate (0.15 µl/min) with an infusion pump (Harvard Apparatus).

### Viruses

Adeno-associated viruses (AAVs) were produced from plasmids courtesy of Dr. Edward Boyden (pAAV-CAG-FLEX-tdTomato; pAAV-CAG-FLEX-GFP) and Dr. Karl Deisseroth (pAAV-CaMKIIa-EYFP). Viral preps were produced and titered (viral genome copies per milliliter – vg/ml) by University of North Carolina Vector Core (CAG-FLEX-tdTomato-AAV5, 4.8 × 10^12 vg/ml; CAG-FLEX-GFP-AAV5, 8.7 × 10^12 vg/ml; CaMKIIa-EYFP-AAV5, 7.1 × 10^12 vg/ml).

### Immunohistochemistry (IHC)

Mice were anesthetized and perfused transcardially with 20 mL PBS and 10 mL freshly prepared 4% PFA. Brains were dissected and postfixed in 4% PFA overnight at 4 °C. Brains were briefly rinsed in PBS, mounted in agarose and 100-µm horizontal serial sections were collected using a Vibratome VT100S (Leica Biosystems). For immunofluorescence staining, sections were then washed in PBS for 5 min under gentle agitation followed by incubation for 1 h in a blocking solution containing 10% goat serum and 0.5% Triton X-100 in PBS. Subsequently, sections were transferred into PBS containing 1% goat serum, 0.01% Triton X-100, primary antibody, and incubated overnight (16–20 h) at 4 °C on a nutating mixer. Primary antibodies used include mouse monoclonal anti-NeuN (Millipore; MAB377, 1:1000), rabbit polyclonal anti-GFP (Invitrogen; A-11122, 1:1000), and rabbit polyclonal anti-Wfs1 (Proteintech; 26995-1-AP, 1:1000). Sections were washed three times with PBS for 5 min each, then incubated in a solution of 1% goat serum, 0.01% Triton X-100, and secondary antibodies for 4 h at 4 °C on a nutating mixer. Secondary antibodies used include goat anti-rabbit Alexa 405 (Thermo Fisher Scientific; A31556, 1:1000), goat anti-rabbit Alexa 488 plus (Thermo Fisher Scientific; A32731, 1:1000), goat anti-mouse Alexa 555 plus (Thermo Fisher Scientific; A32427, 1:1000). Sections were then washed three times in PBS for 5 min each and mounted on microscope slides using Vectashield Plus Antifade mounting media.

### Axon topology fluorescent imaging and analyses

For each viral injected brain, 100 µm horizontal sections were prepared as described for IHC. 6-7 sections (spanning 1.4 mm to 3.6 mm ventral to bregma) were then IHC immunostained for Wfs1. Sections were selected by their highest signal intensity for the injection site and axonal projection targets for analysis. Animals with confirmed precise viral targeting localization for a given target region (PrS or MEC) were selected for analysis and only used if a matched genotype pair (*Adgrl2*^*wt/wt*^*;pOxr1-Cre* and *Adgrl2*^*fl/fl*^*;pOxr1-Cre)* could be established having similar targeting and cell infectivity injection sites. For the MEC, we define proximal MEC as the first third of the region from the PrS going lateral. Distal MEC was defined as the last third and samples selected had the majority of cell bodies within either proximal or distal MEC. Sections were imaged using a Zeiss Axio Imager M2 fluorescence microscope and 2.5x air lens. Acquisition settings were optimized and held constant for each imaged hemisphere of a single brain (ipsilateral and contralateral to injection). Images from P60 animals had regional boundaries added using Wfs1 staining and the rhinal fissure. Fluorescence intensity was subsequently analyzed in ImageJ software using line intensity analysis. After sample selection, images were randomized to blind the analysis and after results were completed, samples were unblinded and groups were compared. Axon topology images were imported into ImageJ for analysis using 25-pixel (layer I projections), 100-pixel (SLM projections) or 200-pixel (PrS/MEC layer III injection targeting) wide line ROIs. These ROIs traversed the proximal to distal axis across the middle of a given region/layer guided by Wfs1 staining to determine unclear regional boundaries. In the MEC, layers were defined as follows; layer I: begins in the end of tissue to where Wfs1 staining is observed. Layer II: rich with Wfs1 staining. Layer III: between the gap of cells which indicates layer IV in the MEC and Wfs1 staining in layer II. To quantify axon fluorescence, measurements were normalized from 0–100% intensity by subtracting the minimum intensity within the sample and dividing by the maximum intensity to give a relative fluorescence value for each sample.

### Single molecule RNA fluorescent in-situ hybridization (smFISH)

P10 or P30 wild type mice were anesthetized with isoflurane and transcardially perfused with 20 mL ice-cold 0.1% DEPC treated and autoclaved PBS, followed by 10 mL 0.1% DEPC treated and autoclaved PFA. Brains were placed into a sterile solution of 4% PFA for 24 h, followed by 10% sucrose overnight at 4 °C. Brains were subsequently cryoprotected by stepwise immersion in sterile 10, 20 and 30% sucrose, remaining in each solution until no longer floating. After 3–4 days in sucrose, brains were mounted on a drop of cryoprotectant and stored at −80 °C until cryosectioning. Brains were cryo-sectioned at 15 µm thickness and adhered to HistoBond+M adhesive microscope slides. After sectioning, tissue in situ hybridization was performed using RNAscope Multiplex Fluorescent RNA probes. Tissue was mounted using Vectashield Plus Antifade mounting media with DAPI and Precision Cover Glass Slips. For single molecule in-situ hybridization, probes included: Oxidation resistance 1 (Mm-Oxr1-C2, 509211; ACDBio) & Reln Reelin (Mm-Reln-C3, 405981; ACDBio). Akoya fluorophores used for hybridization include Opal 620 (C2 channel [Oxr1], FP1495001KT; Akoya Biosciences) and Opal 690 (C3 channel [Reln], P1497001KT; Akoya Biosciences). Imaging was performed on a Zeiss 880 confocal microscope with airyscan using a Plan Apochromat 10x/0.45 M27 air objective scanning in airy fast “Flex” mode (0.7x Nyquist) at 2.17 µs/pixel.

### pOxr1-Cre;Ai14 reporter mice

To assess timing of Cre-recombinase expression, pOxr-Cre mice (C57BL/6N-Tg(Oxr-cre)C14Stl/J) [20] were crossed with tdTomato reporter mice Ai14 (B6.Cg-Gt(ROSA)26Sor tm14(CAG-tdTomato)Hze/J) [21]. After confirming genotypes, brains were collected at regular developmental intervals (P7, P14, P21, P50). Mice were anesthetized and perfused transcardially with 20 mL PBS and 10 mL of fresh 4% PFA. Brains were dissected and postfixed in 4% PFA overnight at 4 °C. Brains were briefly rinsed in PBS before being mounted in 2% agar and sectioned into 100um horizontal sections using Vibratome VT100S (Leica Biosystems). Serial sections were collected from dorsal to ventral (corresponding to bregma −2.36 mm- −3.96 mm in P60 mice) and mounted on microscope slides using Vectashield Plus Antifade mounting media with DAPI and Precision Cover Glass Slips before being imaged. Per each mouse, 8 horizontal sections spanning 200um apart were imaged on a Stereo Investigator V.10.02 (MBF Bioscience) using an Olympus BX51 microscope at 2.5X objective.

### Behavioral analyses

For assessment of animal behavior, sex-matched littermate animals were used from homozygous *Adgrl2*^fl/fl^ parents with or without the presence of pOxr1-Cre allele (*Adgrl2*^fl/fl^ X *Adgrl2*^fl/fl^;pOxr1-Cre^+^). Progeny results in *Adgrl2* deletion in MECIII neurons (MECIII-KO: *Adgrl2*^fl/fl^;pOxr1-Cre^+^) alongside littermate control animals lacking in the pOxr1-Cre allele (*Adgrl2*^fl/fl^). Control and MECIII-KO littermate animals were housed together, and behavioral analysis performed between ages P60-P90.

#### Open field activity

Animals were tested for differences in basal locomotor activity for 5 min in a 60 cm×30 cm arena. Mice were placed in the center of the arena and allowed to freely explore for the duration while being recorded by an overhead camera. The mice were then removed and placed back in their home cage. Videos were analyzed for the first 5 min of recording after holding chamber was lifted using automated video tracking software (Stoelting; Anymaze). Metrics analyzed include total distance moved, average speed, and distance moved in the perimeter. To calculate perimeter distance, the arena was divided into 4 ×8 equal sized sections and the outer 20 sections were classified as the perimeter zone.

#### Novel object recognition task

Mice were tested for recognition memory by placing them in a 60 cm × 30 cm arena with 2 identical objects in opposite corners of the arena (5 cm away from each wall). Mice were allowed to freely roam for 5 min and video recorded for analysis. Mice were then removed and placed back in their home cage for 1 day. One of the objects is switched for a novel object and the mice are given two 5-min trials spaced 1 h apart (the position of the switched object alternates each trial to account for any place preference). Using BORIS video tracking software [22], two double blinded researchers recorded time spent investigating either the familiar or novel object across the 5-min test trial. Results were averaged from the two different researchers for each experiment. Discrimination ratio was calculated = [(time spent with the novel object) – (time spent with the familiar object)] / [[(time spent with the novel object) + (time spent with the familiar object)]. This results in scores ranging from −1 to +1, reflecting preference for novel (>0) or familiar object (<0).

#### Barnes maze spatial memory task

Mice were tested for spatial learning using a modified version of a Barnes maze protocol [23]. The mice are placed on a brightly lit circular platform (92 cm diameter) with 20 holes (diameter 5 cm) equally spaced around the periphery. Only one of the holes has an escape box (7 cm deep, 7 cm wide, 10 cm long), where the other holes have false escape holes placed made of the same texture as the table. Visual cues were present around the outside of the maze to serve as reference to the position of the escape hole. Mice were placed in the center of the maze in a holding chamber for 10 seconds and then lifted so the mice can freely explore. They are assayed for their ability to spatially navigate the maze to the target hole, which they are naturally inclined to seek and avoid the brightly lit open arena. The mice are first given trials where they are guided to the hole until they willingly go in before 120 seconds have elapsed. If over 120 seconds elapse without a hole being entered the trial was excluded and animals were given a ~ 10 min break after which the trial was repeated. One pair of mice was excluded due to inability of one of the mice to complete the task. Mice are trained on four consecutive days with five trials a day (20 trials). Mice are tested 1 day after training and 14 days after for latency to interact with the target hole during a single trial for both tests. Using video tracking software (BORIS), two double blinded researchers independently recorded the latency for mice to reach the target hole and interact with it. Researchers recorded the time in which the mouse was released from their holding container, alongside the time in which the mouse first interacted with the target hole.

#### Water T-maze cognitive flexibility test

Maze consists of a start zone (30 cm length, 10 cm width) and an upper perpendicular zone (70 cm length, 10 cm width) constructed of black plastic and filled with 8 cm of water. For the first swim, one arm of the T-maze was randomly selected by coin flip to be blocked off and a black escape platform was placed on the end of the open arm. Mice were placed in the beginning of the start zone and monitored for time required to reach the escape platform up to 1 min. Trials over 1 min were excluded and after a 10 min break the mice performed another trial. After swim completion, mice were placed in a dry holding chamber for 30 seconds or until dry. During this hold time, the closed arm of the maze was opened and the escape platform switched to the other side. Mice were then placed at the start to complete the second forced swim test, until they reached the second platform. Using video tracking software (BORIS), two double blinded researchers independently recorded the latency for mice to reach the target platform.

### Quantification and statistical analysis

All statistical analysis was performed using Prism 7 software (GraphPad). Group sizes were selected based on previous experience and by using power analysis. Data are shown as mean ± SEM. Normal (Gaussian) distribution was tested for by Shapiro-Wilk normality test prior to subsequent parametric statistical analysis. Significance testing was performed using either one-way ANOVA with post-hoc tukey test for multiple comparisons, or via two-tailed Student’s t-test, as appropriate. Statistically significant differences are indicated by asterisks (*p < 0.05; **p < 0.01; ***p < 0.001). All relevant data presented in this study is available from the authors upon request.

## Results

### Topographically positioned MEC neurons have distinct axonal projection patterns

**Fig. 1:**
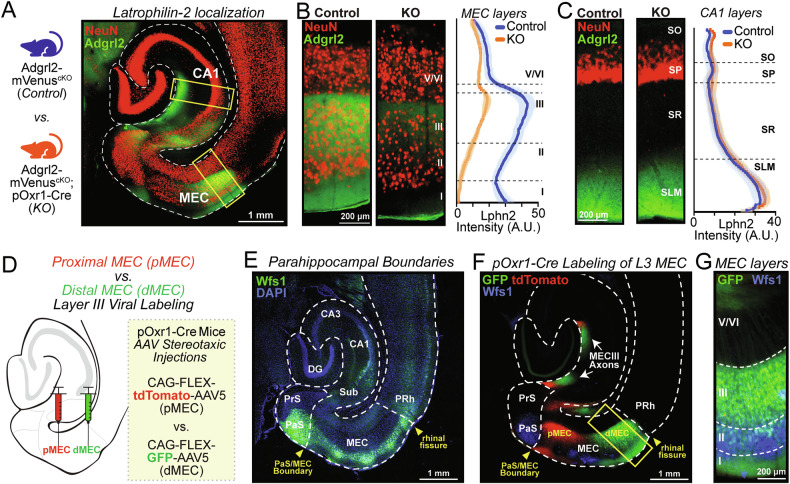
MECIII expressed Latrophilin-2 is topographically organized and associates with distinct circuitry. (**A**) (*Left*) Genotype description of mice used for experiment. (*Right*) representative image of hippocampal circuit with labelled neurons (NeuN) and *Adgrl2* protein. (**B,** **C**) (*Left*) Representative images of *Adgrl2*-mVenus (Control) and *Adgrl2*-mVenus;pOxr1-Cre mice (KO) and (*Right*) line scan of fluorescent intensity measurements across MEC layers (**B**) and CA1 layers (**C**) (n = 4 control/KO mice pairs - 2 male, 2 female; P60). (**D**) AAV experimental schematic to label MECIII neurons in adult pOxr1-Cre mice (P60), targeting proximal MEC (pMEC) using CAG-FLEX-tdTomato-AAV5 and distal MEC (dMEC) using CAG-FLEX-GFP-AAV5. (**E**) Wfs1 IHC labeling for regional boundary delineation of presubiculum (PrS), parasubiculum (PaS), and MEC termination at the rhinal fissure. Yellow arrows indicate landmarks for start and end of the MEC region. (**F**) Representative image of pMEC and dMEC labeled MECIII neurons as described in (**D**), and their axonal projections to the hippocampus (indicated by white arrows). (**G**) Representative image of distal layer III GFP labelled MEC neurons and layer II labeled Wfs1 IHC.

In addition to the *Adgrl2* topographical expression profile that is observed in the MEC, *Adgrl2* expression is confined to select neurons in superficial II/III layers [9]. To test cell-type specific *Adgrl2* dependent circuit assembly, we sought to selectively delete *Adgrl2* from layer III pyramidal MEC neurons (MECIII). To accomplish this, we utilized a transgenic mouse that expresses Cre-recombinase using the Oxr1 promoter that is selectively expressed in MECIII neurons (pOxr1-Cre) [20]. To test for *Adgrl2* protein elimination with this selective Cre-recombinase mediate deletion, we crossed pOxr1-Cre mice with transgenic *Adgrl2*-mVenus^fl/fl^ mice [12]. Due to the current lack of validated antibodies that recognize native *Adgrl2* protein, the *Adgrl2*-mVenus^fl/fl^ mouse strain has two notable features that permits for its detection from specific cell types: (1) Forces the expression, under the control of the native promoter, an *Adgrl2* fusion protein with an integrated mVenus tag. (2) Contains loxP sites flanking the first coding exon, that eliminates all *Adgrl2* expression with Cre-recombinase action. Using these mice, MEC *Adgrl2* is found to be topographically and superficial-layer enriched [9], as well as enriched in the hippocampal stratum lacunosum-moleculare (SLM) [12]. Comparing *Adgrl2*-mVenus mice with or without the pOxr1-Cre allele (Fig. 1A–C), we measured *Adgrl2* immunohistochemical signal in the MEC (Fig. 1B) and hippocampal SLM (Fig. 1C). Doing so, we observe robust decrease in *Adgrl2* signal for MEC sections, with no signal loss in the hippocampal SLM. From this experiment, we can conclude two things. First, *Adgrl2* expression from layer III neurons represents the vast majority of *Adgrl2* protein that is found in the MEC. Secondly, the protein appears to be confined to the dendritic compartment of MECIII neurons, and does not undergo cellular trafficking in MECIII axons that project into the hippocampal SLM.

To confirm the cell-type specific expression for *Adgrl2* deletion as well as testing the use of this genetic tool to investigate differential MEC topographical circuitry, we proceeded to test selective and topographical Cre-recombinase expression in this genetic line. Using pOxr1-Cre mice, we co-labelled proximal and distal MEC neurons with Cre-recombinase dependent GFP (CAG-FLEX-GFP-AAV5) or tdTomato (CAG-FLEX-tdTomato-AAV5) expression AAVs (Fig. 1D). To confirm precise regional boundaries and analyze layer specific expression of the MEC, we utilized an immunohistochemical (IHC) marker for Wolframin Syndrome 1 (Wfs1) [24]. Doing so, we find that Wfs1 IHC labeling is observed strongly throughout the parasubiculum (PaS), and selectively by layer II neurons of the MEC (Fig. 1E). Using Wfs1 IHC to precisely label regional boundaries in pOxr1-Cre injected animals, we observe distinct proximal/distal MEC neuron labelling confined to layer III neurons and not observed in the adjacent regions of the PaS (medial of MEC) or perirhinal cortex (lateral of MEC) (Fig. 1F, G). Secondly, when comparing the projection patterns of layer III labeled neurons from the proximal MEC (pMEC) versus distal MEC (dMEC), we observe spatially distinct axonal organization in the stratum lacunosum-moleculare (SLM) region of the hippocampus (Fig. 1F).

**Fig. 2:**
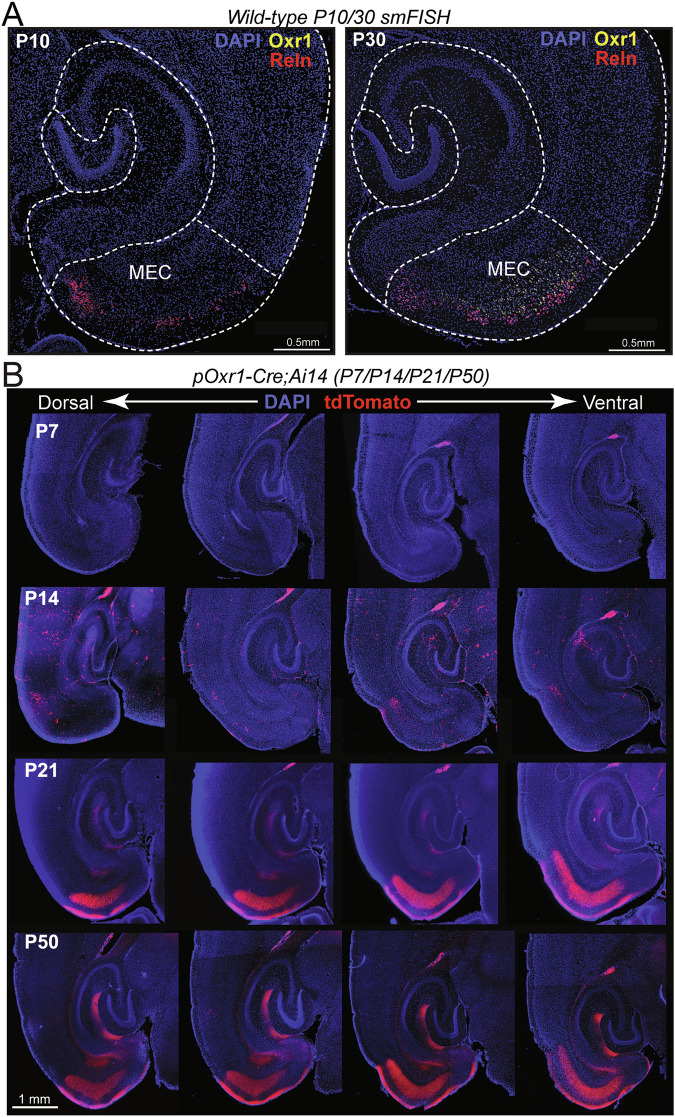
Cre-recombinase expression is postnatally delayed in pOxr1-Cre mice. (**A**) Representative confocal image of P10 (*left*) and P30 (*right*) horizontal brain section smFISH for MECIII marker Oxr1 (yellow) and MECII marker Reln (red). DAPI (blue) fluorescence shown for visualization of all nuclei. (Independent mice: n = 2 (1 female, 1 male) per timepoint) (**B**) Transgenic pOxr1-Cre and tdTomato Cre-reporter mice (Ai14) were crossed, and progeny positive for both alleles (pOxr1-Cre;Ai14) were analyzed during weekly timepoints during development (P7/P14/P21) and compared to adult (P50) tdTomato expression. Shown are representative images of multiple horizontal serial sections along the dorsal to ventral axis (Independent mice: P7 n = 3 (2 female, 1 male); P14 n = 2 (1 female, 1 male); P21 n = 4 (3 female, 1 male); p50 n = 1 male).

After this confirmation of selective MECIII expression of Cre-recombinase in pOxr1-Cre mice, we next set out to check for the developmental timing of its expression. We began by surveying the expression of Oxr1 itself. Examining Oxr1 mRNA using single molecule fluorescent in situ hybridization (smFISH) at two postnatal developmental timepoints (P10 and P30), we found that there is a noticeable developmental delay in the expression of Oxr1 transcripts (Fig. 2A). While Oxr1 signal is not observed in young (P10) animals, robust expression is observed after adult maturation (P30). This suggests that there would be a similar developmental delay in Cre-recombinase expression in pOxr1-Cre mice. To confirm the timing of Cre-recombination in these mice, we crossed pOxr1-Cre animals with the reporter line that expresses tdTomato in the presence of Cre-recombinase (Ai14) [21]. Examining pOxr1-Cre^+^;Ai14^+^ mice at postnatal timepoints at regular intervals (P7, P14, P21, P50), we find there to be a developmental delay in the expression of the tdTomato Cre-reporter (Fig. 2B). In pOxr1-Cre^+^;Ai14^+^ mice, tdTomato expression is largely undetectable at P7. At P14 timepoint, there is evidence of sparse MECIII neuron labeling, alongside infrequent non-specific cellular labeling outside the MEC. By postnatal day 21 however, robust MECIII neuron labeling is observed in their cell bodies, as well as their axon projections to the hippocampus. This labeling is stable thereafter and is similarly observed in adults (P50). Therefore, timing of Cre-recombinase expression appears to be developmentally delayed, with full expression turning on postnatally between P14-21. Taking these experiments together, we validate the use of pOxr1-Cre mice as a genetic tool to evaluate the role of *Adgrl2* in MECIII neurons, with the caveat that Oxr1 promoter mediated Cre-recombinase expression and *Adgrl2* deletion is developmentally delayed.

### Adgrl2 deletion in MEC layer III neurons does not affect hippocampal projection topology

**Fig. 3:**
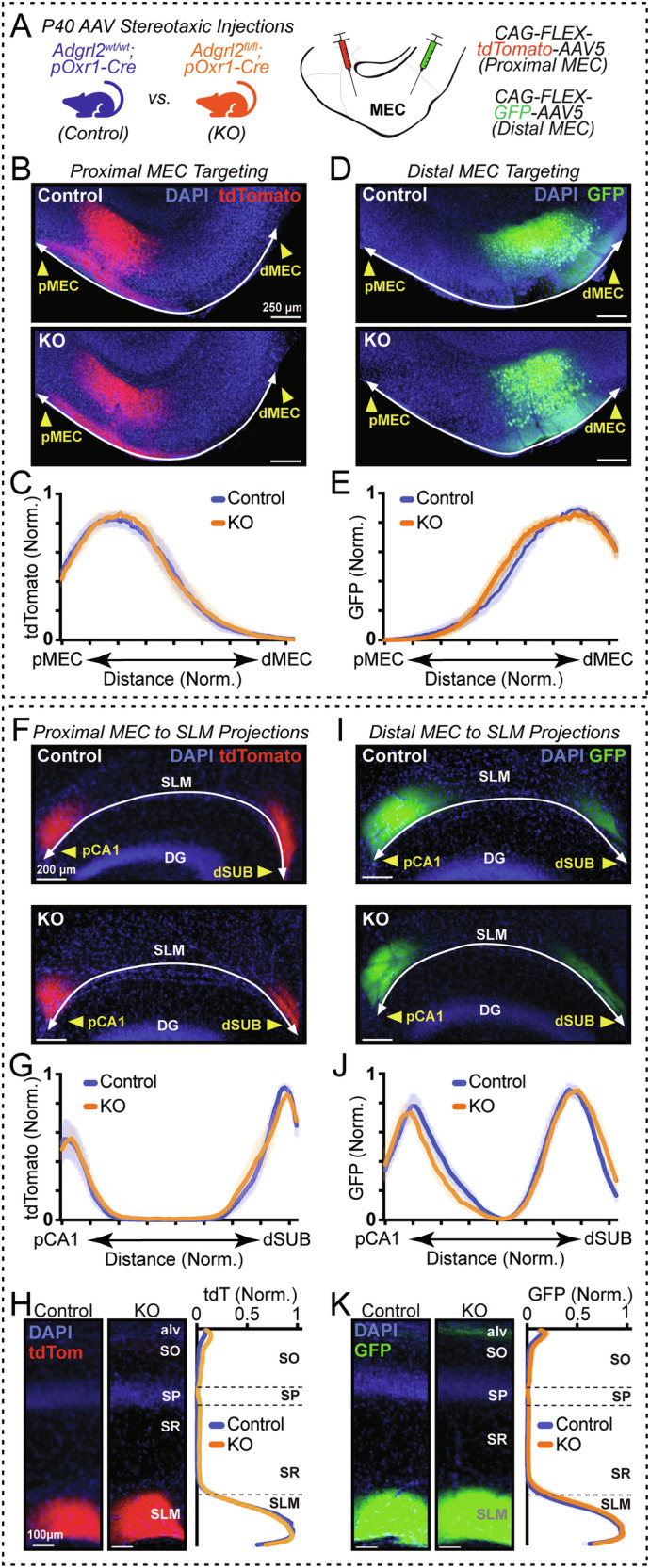
*Adgrl2* deletion in MEC layer III neurons does not affect axon projection topography to the hippocampus. (**A**) Experimental design. AAV injections targeting proximal and distal MEC performed in control and MECIII *Adgrl2* knockout (KO) animals at P40, imaging at P60. (**B**) Representative image of tdTomato labelled proximal MEC neurons for control (top) and KO (bottom) mice. (**C**) Normalized line intensity scan measurements across the proximal to distal MEC layer 3 for control and KO animals (**D,** **E**) Similar as for B-C, except for distal targeted MECIII neurons labeled with GFP expressing AAVs. (**F**) Representative image of control and KO tdTomato labelled axons in the hippocampal SLM. (**G**) Line intensity scan measurement across the proximal CA1 (pCA1) to distal subiculum (dSUB) axis in the SLM for control and KO animals. (**H**) Line intensity scan measurement across CA1 layers comparing layer specific targeting of tdTomato axons for control and KO. Plots and summary graphs shown are means +/− SEM (n = 6 control - 4 male, 2 female; n = 6 KO - 2 male, 4 female). (**I**–**K**) Similar as F-H, except for distal MECIII neurons axonal projections in the hippocampus visualized by GFP. Plots shown are means +/− SEM (n = 6 control - 4 male, 2 female; n = 6 KO - 3 male, 3 female).

After confirming selective Cre-recombinase expression in MECIII neurons in pOxr1-Cre mice, we next set out to survey *Adgrl2* function in these neurons for controlling both input and output axonal targeting, to and from the MEC. To do so, we crossed pOxr1-Cre mice with *Adgrl2* conditional knock-out mice that have loxP sites flanking the first coding exon of *Adgrl2* (*Adgrl2*^fl/fl^) [12]. The resulting transgenic mouse line exhibits selective deletion of *Adgrl2* in layer III neurons of the medial entorhinal cortex (*Adgrl2*^fl/fl^;pOxr1-Cre). As a control, we used animals with native *Adgrl2* alleles not impacted by Cre-recombinase expression (*Adgrl2*^wt/wt^;pOxr1-Cre). Using these animals, we performed proximal/distal MECIII neuron labeling with Cre-recombinase dependent tdTomato and GFP expression AAVs (Fig. 3A). We first quantified injection localization between *Adgrl2*^wt/wt^;pOxr1-Cre (Control) and *Adgrl2*^fl/fl^;pOxr1-Cre (MECIII-KO) mice by measuring relative fluorescence levels across layer III from the beginning of proximal MEC (pMEC; marked at the PaS border with immunolabelled Wfs1), through the distal MEC (dMEC; marked by the rhinal fissure and decrease in prominent Wfs1 labeling). While quantifying viral localization across the MEC in this fashion, we compared samples with similar localization and infectivity between genotypes for both tdTomato labeled pMEC (Fig. 3B, C) and GFP labelled dMEC (Fig. 3D, E).

With similar populations of neurons labeled in control and MECIII KO mice, we then proceeded to compare the targeting of their main axonal projection pathway, the hippocampal stratum lacunosum-moleculare (SLM) (Fig. 3F–K). In this analysis, we tested the genetic manipulation of *Adgrl2* in a purely presynaptic MECIII fashion, as hippocampal cells do not express Cre-recombinase in pOxr1-Cre mice [20]. For this analysis, the MECIII axons that project in a topographical patterning to the hippocampus was quantified within the SLM layer from the CA1 region proximal to CA3 (pCA1) to the distal subiculum (dSUB) region ending at the presubiculum (PrS) border. By comparing relative fluorescent intensity measurements in this fashion, we observe no difference between control and MECIII-KO mice for axon topographical organization in the SLM (Fig. 3F, G). Additionally, we measured SLM layer specific targeting in CA1 by measuring fluorescence intensity across hippocampal layers from the alveus through the SLM. Comparing CA1 layer specificity of MECIII axon targeting between genotypes, no alterations in SLM targeting was observed (Fig. 3H). This analysis was repeated for dMEC injections, with similar findings observed with SLM projection topology (Fig. 3I, J) and layer specificity (Fig. 3K). In addition to these MECIII projections to the ipsilateral SLM, we observed strong projections as well to the contralateral SLM as similarly described [9, 25]. Performing similar analysis on these same labeled MECIII inputs for contralateral SLM projections, we again observed no differences between genotypes (data not shown). Collectively, it appears that neither proximal nor distal MECIII neurons require *Adgrl2* for their spatial axon targeting to the hippocampus.

### Adgrl2 deletion from MEC layer III neurons alters contralateral MEC projection topology

**Fig. 4:**
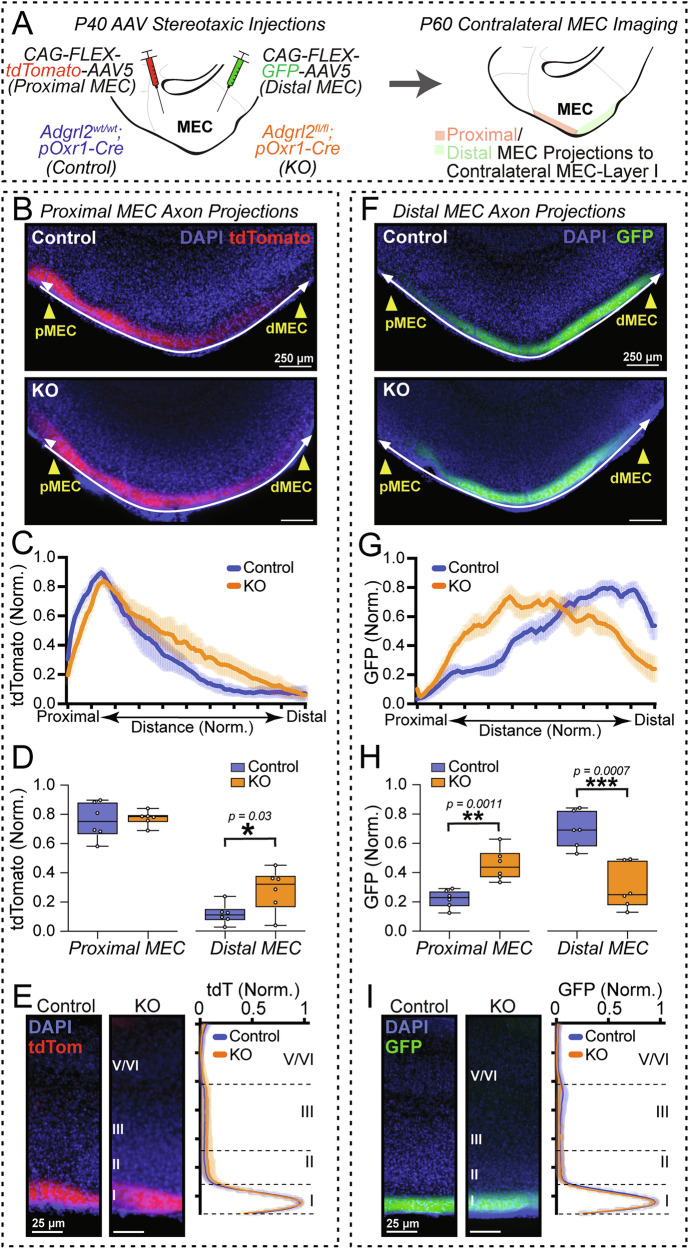
*Adgrl2* deletion from MEC layer III neurons alters contralateral MEC input topology. (**A**) Experimental design. AAV injections targeting proximal and distal MEC performed in control and MECIII *Adgrl2* knockout (KO) animals at P40. Projections to the contralateral MEC layer I are imaged at P60 and analyzed. (**B**) Representative image of tdTomato labelled proximal MEC axons in contralateral MEC for control (top) and KO (bottom) mice. (**C**) Line intensity scan measurements across the proximal to distal axis in MEC layer 1 comparing axon localization between control and KO. (**D**) Summary graph of averaged fluorescence levels for control and KO at proximal (distance 15–25%) and distal (distance 75–85%) positions. (**E**) (*Left*) Representative image of control and KO contralateral proximal MEC labelled axons in the MEC layer I. (*Right*) Line intensity scan measurements across the indicated proximal MEC layers. Shown are normalized fluorescent intensity measurements across MEC layers from deep (layer V/VI) to superficial (layer I) comparing layer specific targeting of tdTomato axons for control and KO. Plots and summary graphs shown are means +/− SEM (n = 6 control - 4 male, 2 female; n = 6 KO - 2 male, 4 female). (**F**–**I**) Similar as for B-E, except for contralateral distal MEC projections. Plots and summary graphs shown are means +/− SEM (n = 6 control - 4 male, 2 female; n = 6 KO - 3 male, 3 female). Statistical analyses for summary graphs was performed using Student’s t-test (*p < 0.05, **p < 0.01, ***p < 0.001).

In addition to the well-known MEC projections into the hippocampus, we also observed prominent MECIII axons that project to the contralateral MEC as previously described [9, 26]. Similarly as with MECIII axon projections to the hippocampus, these MECIII projections to contralateral MEC appear to be both topographically organized and layer targeted (Fig. 4A). Neurons from pMEC target the contralateral pMEC, whereas dMEC neurons target contralateral dMEC. In both projections, MECIII axons appear to be targeted exclusively to contralateral MEC layer I. With this clear topographical and layer organization, we next explored the impact *Adgrl2* MECIII neuron deletion would have on the specificity of this patterning. To compare the topographical projection patterns from pMEC tdTomato labelled MECIII axons into MEC layer I, line scan quantification was performed traversing the proximal to distal axis across MEC layer I as described previously (Fig. 3). In doing so, a slight shift is observed in the axonal targeting for *Adgrl2* deletion in MECIII neurons, with a small increase in axonal targeting observed in the dMEC compartment (Fig. 4B–D). Lastly, we analyzed the layer specific targeting for these pMECIII axon projections. Performing a line scan analysis of the contralateral MEC through layers I-VI, we observed layer I specific targeting that remains intact regardless of *Adgrl2* MECIII status (Fig. 3E). We then repeated this topographical and layer targeting analysis for dMECIII neurons and their projections to contralateral MEC (Fig. 4F–I). For these projections, we found a more robust shift in the axon topological targeting to *Adgrl2* MECIII deletion (Fig. 4F–H). For these *Adgrl2* deleted dMECIII projections, rather than projecting strongly to the contralateral dMEC as normally observed, these axons appear to shift their targeting more proximally within the MEC (Fig. 4G, H). Layer- specific targeting however, appears to be *Adgrl2* independent (Fig. 3I). Altogether, we found that *Adgrl2* is essential for precise dMECIII axon targeting to contralateral dMEC in terms of topographical patterning, while simultaneously serving as a repulsive signal for pMEC projections to contralateral dMEC.

### Adgrl2 deletion from MEC layer III neurons alters PrS to MEC topology

**Fig. 5:**
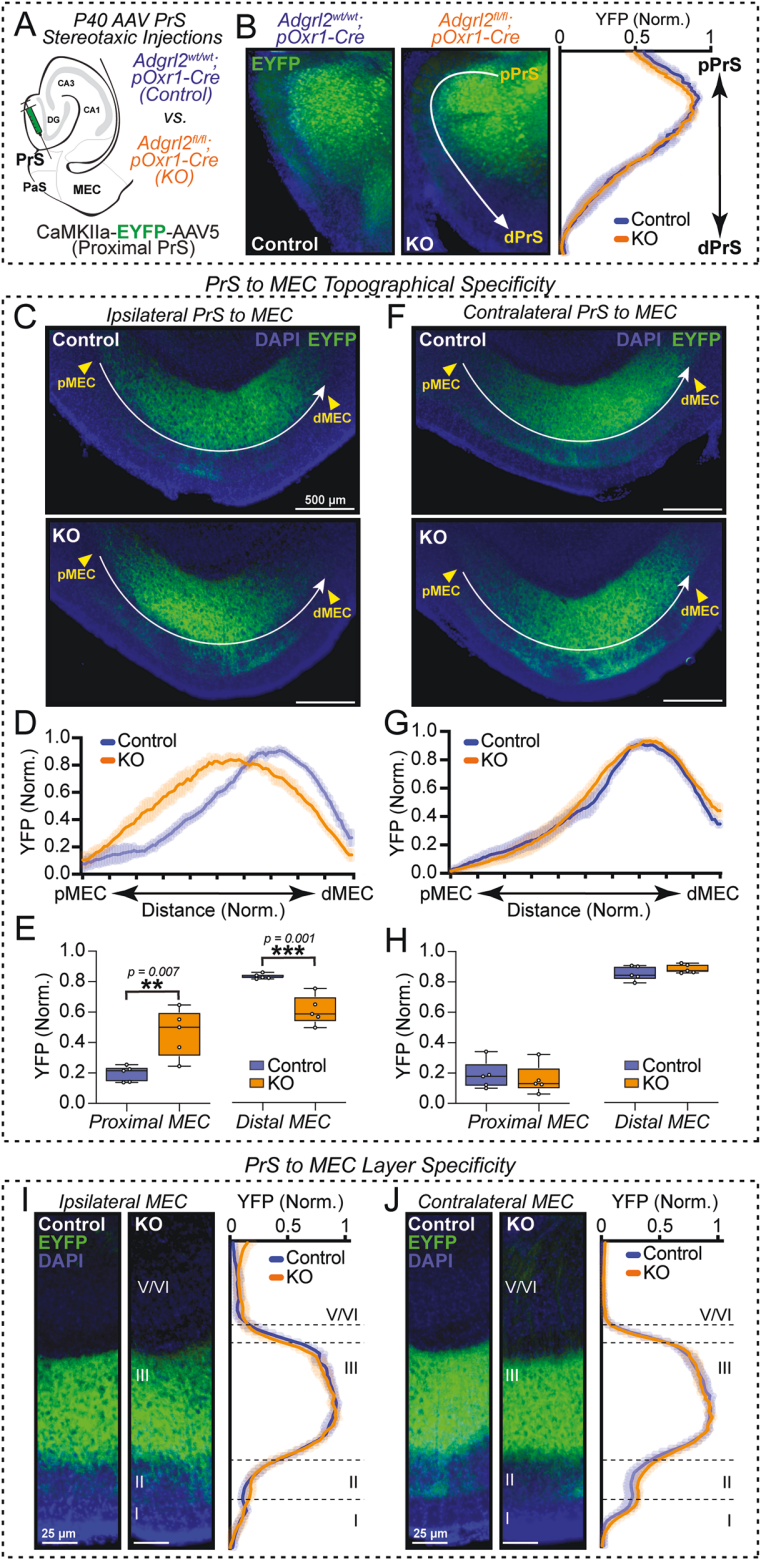
*Adgrl2* deletion from MEC layer III neurons alters presubiculum input topology. (**A**) AAV experimental schematic targeting PrS using CaMK2a-EYFP-AAV5 to label proximal presubiculum neurons. (**B**) (Left) Representative image of YFP labelled proximal PrS neurons for control and KO mice. (Right) Line intensity scan measurement across the proximal to distal PrS layer 3 comparing localization of injection for control and KO. (**C**) Representative image of YFP labelled PrS axons in ipsilateral MEC for control (top) and KO (bottom) mice. (**D**) Line intensity scan measurement across the proximal to distal MEC layer III comparing axon localization between control and KO. (**E**) Summary graph of averaged fluorescence levels for control and KO at proximal (distance 15–25%) and distal (distance 75–85%) positions. Statistical analyses were performed using Student’s t-test (**p < 0.01, ***p < 0.001). (**F**–**H**) Similar as C-E, except for the contralateral MEC. (**I**) (Left) Representative image of control and KO PrS labelled axon projections into the ipsilateral MEC layer III. (Right) Line intensity scan measurement across MEC layers comparing layer specific targeting of PrS axons for control and KO. (**J**) Similar as I, except for contralateral MEC. Plots and graphs shown are means +/− SEM (n = 5 control – 3 male, 2 female; n = 5 KO - 3 male, 2 female).

Previously, we found that by using retrograde neuron labeling, deletion of *Adgrl2* in one MEC hemisphere leads to a selective impairment in connectivity from ipsilateral PrS to MEC using retrograde tracers [9]. With the majority of *Adgrl2* protein in the MEC being genetically removed in pOxr1-Cre mice (Fig. 1B), we hypothesized that this cell type is critical for the topographical organization of PrS to MEC connections. To test this hypothesis, we utilized *Adgrl2*^wt/wt^;pOxr1-Cre (Control) and *Adgrl2*^fl/fl^;pOxr1-Cre (MECIII-KO) mice as before. We then labelled proximal PrS neurons and their projections in these animals with stereotaxic targeted viral injects of EYFP expression AAVs (CaMKIIa-EYFP-AAV5) (Fig. 5A). In this experiment, genetic deletion of *Adgrl2* is purely in postsynaptic MECIII neurons, as presubiculum neurons do not express Cre-recombinase in pOxr1-Cre mice [20]. Comparing the injection localization/expression of EYFP, we analyzed animals with similar PrS targeting (Fig. 5B). We then proceeded to compare the projection patterning of their outputs into the MEC layer III for both brain hemispheres (Fig. 5C–H). Analyzing the PrS axon organization in the MEC, we find proximal PrS targeted the dMEC layer III on both hemispheres as previously reported [4, 9]. When comparing control and MECIII-KO mice however, there is a clear shift in this PrS topographical targeting to the ipsilateral MEC with MECIII *Adgrl2* deletion (Fig. 5C–E). Interestingly, when similarly analyzing the PrS projections to MEC on the opposite hemisphere, this long-range topographical targeting appears intact (Fig. 5F–H). Lastly, when comparing the layer specificity of PrS axons to the MEC, both ipsilateral and contralateral projections exhibited similar layer III targeting specificity (Fig. 5I, J). Together, this implicates *Adgrl2*-MECIII neurons to be selectively controlling short-rage ipsilateral PrS to MEC connections for their topographical, but not layer specified targeting. Long-range contralateral PrS to MEC input targeting on the other hand, appears to be *Adgrl2* independent.

### Adgrl2 deletion in MECIII neurons impairs spatial-sequence learning

**Fig. 6:**
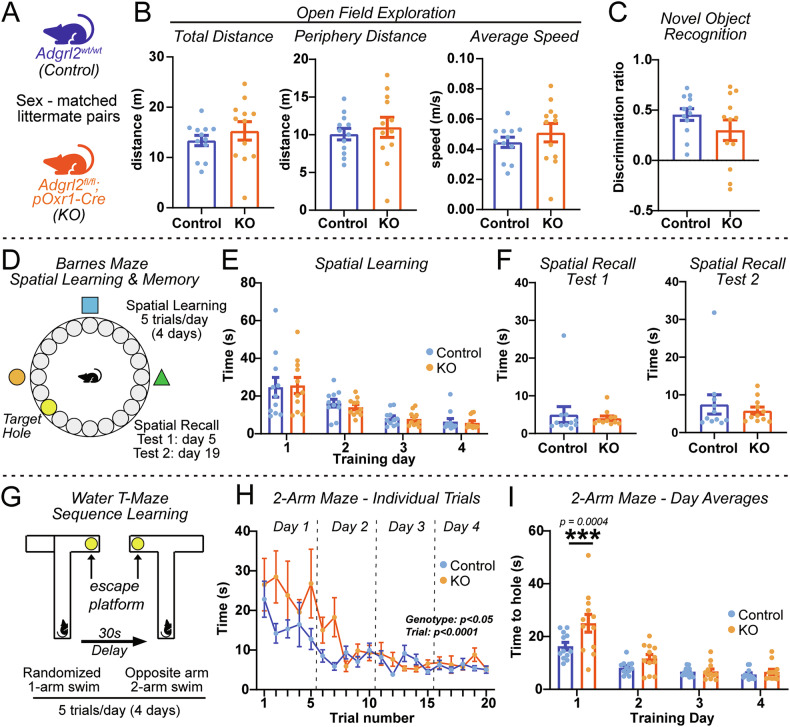
*Adgrl2* deletion from MEC layer III neurons impairs reversal spatial learning. (**A**) *Adgrl2*^fl/fl^ mice were crossed with *Adgrl2*^fl/fl^;pOxr1-Cre mice to generate offspring that were homozygous *Adgrl2*^fl/fl^ without (control) or with the pOxr1-Cre allele (KO). Sex matched control/KO pairs were used for subsequent behavior experiments. (**B**) Summary graphs of open field locomotor/exploration test. (Left) total distance traversed during the open field task, (middle) distance traversed in the periphery of the open field, (right) average speed across the open field task. (**C**) Summary graph of novel object discrimination. (**D**–**F**) Barnes maze spatial learning/memory tests. (**D**) Barnes Maze experimental design. Mice were trained 5 times a day for 4 days with memory test 1 the following day after training and memory test 2 at 14 days after the final training. (**E**) Summary graphs of maze training. Plotted is average time spent to find the target hole across 5 training trials conducting on days 1–4. (**F**) Summary graphs of time spent to find target hole on memory test 1 (left) and test 2 (right). (**G,** **H**) Alternating water T-maze cognitive flexibility task. (**G**) Experimental schematic. Mice were trained 4 days with individual trials consisting of a 2-swim protocol (5trials/day). First swim consists of randomized placement of escape platform in either left or right arm of a 1-arm maze. Second swim occurs after a 30 s delay, with mice exposed to an opened 2-arm open maze with escape platform placed on opposite arm from the first swim. (**H**) Summary plots of time to platform during 2-arm swim test trials. Statistical analyses were performed using repeated-measure ANOVA with post hoc comparisons, finding learning effect by trial (p < 0.0001) and between genotypes (p = 0.014). (**I**) Summary graphs of time to platform averaged across 5 trials/day during 2-arm swim test. Plots and graphs shown are means +/− SEM. Statistical analysis were performed using Student’s t-test (***p < 0.001). n = 12 control/KO mice pairs (3 male, 9 female pairs), P60-90.

To examine whether *Adgrl2* function in MECIII neurons affects animal behavior, we next sought to test if this genetic manipulation impacts entorhinal cortex dependent episodic learning and memory. Previous work that targeted *Adgrl2* deletion to the CA1 hippocampal region, was found to result in learning deficits of spatial tasks that involve a temporally separated sequence of events (i.e. water T-maze), but no effect on tasks that purely assess spatial learning and memory (i.e. novel object recognition, novel area exploration, Barnes maze) [12]. We then asked, is MECIII *Adgrl2* expression similarly required for temporal/spatial sequence learning? To test this hypothesis, we set out to perform a variety of entorhinal cortex dependent behaviors alongside non-entorhinal dependent learning tasks using sex matched littermate pairs of control and MECIII *Adgrl2* deleted mice (Fig. 6A). First, we measured novel area exploration with a 5-min open field test, dividing the arena into central and peripheral zones with behavior tracking software. We analyzed movement speed, distance, and periphery distance to determine if there were differences in locomotor activity in these mice, or anxiety like behavior indicated by an increase of time spent in the periphery of the arena [27]. We did not observe any significant differences in distance traversed, distanced traversed in the periphery, or movement speed of the mice due to genotype (Fig. 6B). Next, we performed a novel object recognition task, implicated as dependent on the hippocampal dentate gyrus and lateral entorhinal cortex [28, 29]. Using a discrimination ratio to compare time spent with novel and familiar objects 1 h after exposure to familiar objects, we did not find significant differences between groups due to genotype (Fig. 6C). Third, we proceeded to test spatial learning and memory, by performing a Barnes maze behavior analysis [23]. We subjected these same mice to the Barnes Maze where they are placed in a brightly lit platform containing 20 evenly spaced closed holes around the perimeter (Fig. 6D). Visual cues were placed outside of the maze to help orient the mice, and one of the platform holes (target) was opened to provide a darkened space under the platform. For 4 days, mice were tested 5 times each day and measured for their ability to find the target hole at a fixed location. On day 5, a single trial (test 1) was performed to examine short term memory. Mice were then home cage housed for 2 weeks, after which another single trial was performed to assess long term spatial memory (test 2). With these experiments, we found no changes in learning the task (Fig. 6E), nor in the short-term or long-term memory recall test trials (Fig. 6F). Lastly, we then performed a spatial task that assesses animal ability to be cognitively flexible and learn sequences of spatial events, the water t-maze test (Fig. 6G). This assay incorporates an alternating spatial task with two swim trials separated by a 30 second time delay in which the mice must learn the escape platform on the second trial is always on the opposite side of the first randomized trial (Fig. 6G). In performing this assay, we find that *Adgrl2* MECIII deleted mice exhibited a longer latency to find the escape platform upon reversal in the 2-arm maze during the first day of training but not subsequent training days (Fig. 6H, I). This suggests that MECIII deletion of *Adgrl2* impairs cognitive flexibility learning rates, but animals are able to retain these spatial-sequence memories once the task is acquired. Altogether, these behavioral assays indicate *Adgrl2* MECII deletion results in a selective impairment in spatial/temporal sequence learning tasks that requires cognitive flexibility, while other entorhinal cortex dependent tasks are unaffected.

## Discussion

In this study we investigated the role of *Adgrl2* in MECIII neurons at multiple levels, from animal behavior to topographic circuit assembly. During analysis of animal behavior, our results revealed largely intact episodic learning and memory encoding with subtle impairments due to *Adgrl2* MECIII deletion. Specifically, we find deficits in the learning rate of a spatial task that require sequence pattern recognition, the water T-maze. Interestingly, this same water T-maze sequence learning task impairment was also found with targeted deletion of *Adgrl2* from hippocampal CA1 neurons [12]. Thus, *Adgrl2* appears to be involved on both sides of the interconnected MEC-CA1 circuit to support spatial sequence learning. Together, these findings point towards a model of episodic learning and memory that involves distinct circuit pathways that encode spatial versus non-spatial information. In the hippocampus, it has been proposed that proximal hippocampal CA1 has been found in both rodents and humans to be highly tuned to spatial information, whereas distal CA1 is sensitive to temporal information [30, 31]. In similar fashion, it appears that *Adgrl2* is organized in entorhinal-hippocampal circuitry that is responsible for temporal aspects of episodic learning and memory encoding.

**Fig. 7:**
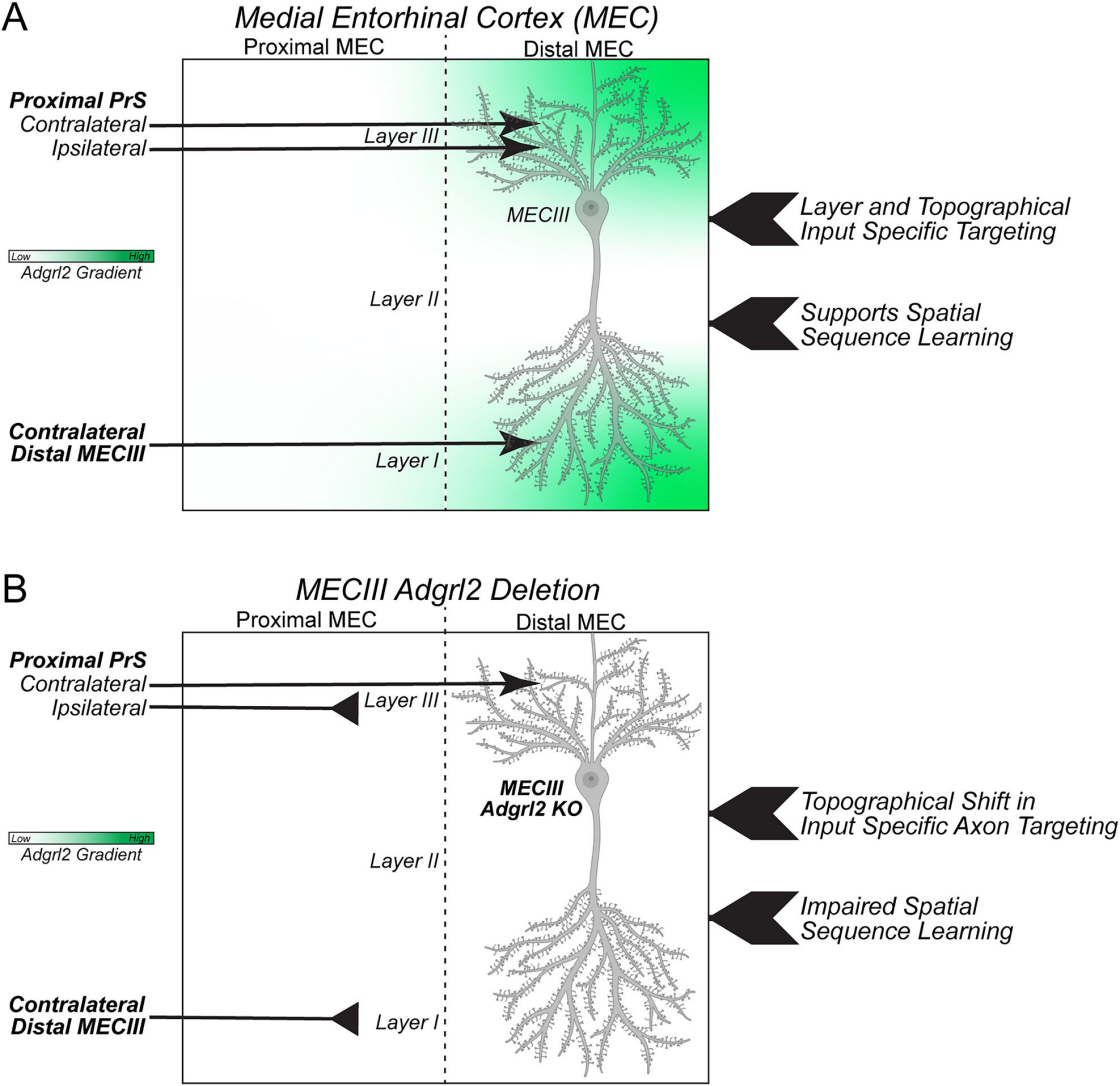
Model for *Adgrl2* function in MECIII neurons. (**A**) MECIII expressed *Adgrl2* is organized in a topographical and layer specific fashion that overlaps with input specific axon targeting. Distal MEC inputs include projections from the ipsilateral/contralateral Proximal PrS that target MEC layer III, and contralateral distal MECIII projection neurons that target MEC layer I. (**B**) MEC circuitry impacts upon MECIII *Adgrl2* deletion include targeting remodeling of select inputs arising from the ipsilateral proximal PrS and contralateral distal MECIII neurons.

In our studies on topographic circuit assembly, we correspondingly found select functions for *Adgrl2*. Specifically, MECIII *Adgrl2* deletion led to axon mistargeting from ipsilateral PrS and contralateral MECIII inputs (Fig. 7). Contralateral MECIII axons that target layer I MEC in a topographic manner shifted more medial with MECIII *Adgrl2* deletion without an effect on layer targeting. Likewise, ipsilateral PrS axons that target layer III of the MEC, required MECIII *Adgrl2* expression for topographical, but not layer specific targeting. Interestingly, *Adgrl2* expression is observed in both pre- and postsynaptic neurons of these interconnected regions [9], which raises the possibility that *Adgrl2* may be functioning in both pre- and postsynaptic compartments for controlling connectivity. In this study however, we find evidence that *Adgrl2* appears to have clear postsynaptic, but not presynaptic function in controlling input specific axon targeting. In a purely presynaptic genetic manipulation, we found that the axon topographical and layer specific targeting to the hippocampal SLM is unaffected by *Adgrl2* deletion in MECIII neurons. Therefore, the axon guidance mechanisms for MECIII neurons to the hippocampus appears to be independent of *Adgrl2* expression. In a mixed presynaptic and postsynaptic *Adgrl2* deletion context however, MECIII to contralateral MEC layer I projections were topographically shifted to more proximal MEC targeting. While we are not able to dissect out the presynaptic versus postsynaptic effects with this targeting, we were able to perform a purely postsynaptic genetic manipulation with our studies on PrS to MEC connections. In these experiments, *Adgrl2* expression is intact in PrS neurons, and deleted in MECIII neurons. In analyzing PrS axon projections to the MEC, we see clear topographical targeting that is dependent on postsynaptic *Adgrl2* MECIII expression. Perhaps one of the most interesting observations from this study, is the hemisphere dependency for axon targeting. With our PrS axon analysis, we find that only ipsilateral PrS projections to the MEC were topographically shifted, while contralateral targeting was unaffected. This suggests that clear differences in hemisphere dependent axonal targeting mechanisms are at work for PrS to MEC connectivity. While the genetic programs that instruct ipsilateral/contralateral specific circuit assembly is currently not well understood in entorhinal cortex circuits, there is evidence in other circuit systems for hemisphere dependent genetic programs. This includes the visual system where retinal ganglia cell populations that project ipsilaterally versus contralaterally are implicated to differentially express >300 genes during the critical period of axonal outgrowth [32], and in motor systems where ipsilateral versus contralateral projecting precerebellar pontine nucleus neurons are implicated to possess differential gene regulatory programs controlled by transcriptional factors Nhlh1 and Nhlh2 [33]. This study introduces a new molecular player that participates in hemisphere dependent circuit assembly. In the PrS to MEC circuit, *Adgrl2* MECIII expression is essential for correct ipsilateral connections. Contralateral PrS to MEC circuit connectivity is *Adgrl2* independent, which suggests that *Adgrl2* is either not involved, or there is built in redundancy to ensure proper axon targeting. Since PrS projections are required to traverse a greater distance with greater navigation complexity to the contralateral MEC than for ipsilateral MEC, an increased number of molecular components are likely part of the differential genetic program that specify contralateral axon targeting.

At the molecular level, Latrophilins interact with extracellular binding partners including Teneurins and FLRTs, to form large cellular-adhesion complexes and promote synapse development [10, 11, 17, 34–36]. Although these proteins can form macromolecular interactions, it is not clear which complexes are stable at synaptic sites and in which pre- or postsynaptic compartments they are presented. For example, *Ten3* and *Adgrl2* extracellular interaction has been demonstrated to have a repulsive signaling effect for axon guidance and synaptogenesis rather than an attractive one to organize CA1 to subiculum hippocampal circuit assembly [8]. A question that arises from our work, if *Adgrl2* + PrS axons are normally repulsed by *Ten3* in proximal MEC, would they not still be repulsed by *Ten3* if *Adgrl2* is lost in the MEC? The assumption that *Adgrl2*+ axons from the PrS are repulsed by *Ten3* in the MEC is dependent on *Adgrl2* having a presynaptic function in PrS. Yet, it is unclear what function *Adgrl2* has in the axon. Our data suggests that *Adgrl2* does not have a presynaptic function in MECIII neurons for axon targeting. Its role on the postsynaptic side however is clear, with *Adgrl2* in MECIII neurons serving as an attractive cue for incoming PrS axons. When MECIII *Adgrl2* is lost, PrS axons become mistargeted. In an opposite pattern, *Ten3/4* deletion has been shown to have presynaptic, but not postsynaptic function in controlling MEC to hippocampal CA1 circuit development [37]. This further adds to a working model that these molecular players have defined input-output circuit building roles, with teneurins and latrophilins functioning on opposite sides of the synapse. Our full understanding of the latrophilin extracellular to intracellular signaling mechanisms at work during circuit assembly however, is incomplete. Mutating the extracellular domains responsible for teneurin or *FLRT* binding, has been shown to disrupt *Adgrl3* dependent synapse assembly [11]. Intracellularly, *Adgrl2* and *Adgrl3* postsynaptic roles for synaptic formation has likewise been shown to be dependent on its ability to signal as a GPCR [38]. In axon guidance processes however, *Adgrl2* function on the postsynaptic side as a repulsive signal for Ten3 expressing axons, has been shown to be independent of its GPCR signaling [39]. Yet, *Adgrl2* misexpression in presynaptic neurons can lead to axon mistargeting, which is dependent on its GPCR signaling [39]. To fully understand the signaling mechanisms underlying latrophilin dependent axon guidance, will require characterizing the complete set of *Adgrl*-containing complexes involved in both the attraction of incoming axons and stabilization of synaptic sites. Equally, identifying and understanding the *Adgrl*-containing complexes that mediate synaptic rejection and axon repulsion is essential for a complete picture of their roles in neural circuit assembly.

Complicating the interpretation of our results, are the limitations of the mouse model used. An intriguing finding during this work is the developmental delay of *Adgrl2* deletion ( > P14) found in the *Adgrl2*^fl/fl^;pOxr1-Cre mice. Despite this delay in *Adgrl2* MECIII deletion, the spatial organization of select inputs into the MEC are impacted. Are axons still targeting during this time? While the timing of axonal targeting from these sources is unclear, maturation of the entorhinal cortex has previously been found to be established in steps that are layer organized. Cellular maturation has been found to begin with layer II stellate cells that reach maturity ~P14, before progressing to layer V at ~P30 [40]. Consistently, our analysis indicates that cellular maturation of MECIII neurons does not occur until P14-21, as indicated by the delay in mRNA expression of transcripts found in adults (e.g. Oxr1). As such, layer III neural circuitry is likely still under development during this time. Alternatively, it is an open possibility that *Adgrl2* MECIII expression may instead be required for the maintenance of axonal positioning. There is growing evidence that the adult brain is capable of more extensive remodeling of neural circuits not limited to synaptic plasticity, that includes the retraction and/or growth of axons [41]. The delayed deletion of MECIII *Adgrl2* in *Adgrl2*^fl/fl^;pOxr1-Cre mice during postnatal development opens up two major questions of the input remodeling observed in this study: (1) Do PrS axons fail to target appropriately to the MEC during development? Or alternatively, (2) Do PrS axons project appropriately to the MEC, but then fail to stabilize in the absence of MECIII *Adgrl2*? To answer these possibilities, examining *Adgrl2*^fl/fl^;pOxr1-Cre mice at earlier developmental time points would be required. The ability to specifically label inputs from the PrS at earlier timepoints however, is not currently technically feasible given the small size of the PrS prior to maturation to allow for precise stereotaxic labeling during development. To be able to discern between these possibilities, would require the development of genetic tools to allow for PrS specific labeling. As such, investigating the timing of PrS-MEC neural circuit development and *Adgrl2* functional role in this context remains an open question.

Insight into circuit remodeling impacts that occur with *Adgrl2* MECIII deletion however, might also come from examining the postsynaptic side. Previously, we have found that early postnatal *Adgrl2* deletion (P0) in MEC layer III neurons leads to loss of apical dendritic spine structures, major sites of excitatory synaptic connections [9]. While it is unclear how later loss of *Adgrl2* affect MEC layer III pyramidal neuron dendritic spine development in this study, there is previous evidence that latrophilins function not only during development but also for maintenance of spine structures. In hippocampal CA1 neurons, *Adgrl3* deletion results in the loss of spine structures and synaptic transmission in the Stratum radiatum, which is equally negatively impacted with timed *Adgrl3* deletion at both early postnatal development (P0) and at adult timepoints (P21) [11]. Similarly, we would expect PrS to MECIII connections to be negatively impacted regardless of the timepoint of *Adgrl2* MECIII deletion. Further, MECIII expression of *Adgrl2* is found to be enriched during both early postnatal (P10) and matured MECIII neurons (P30) [9], suggesting that developmental and maintenance functions of *Adgrl2* are of equal importance. However, we can not conclusively say if earlier deletion of *Adgrl2* from MEC layer III pyramidal neurons would dramatically alter the circuit remodeling that is observed in *Adgrl2*^fl/fl^;pOxr1-Cre mice.

In closing, given the complexity of MEC input/output circuitry with its topographical and layer specific organization, distinct molecules are likely involved for directing unique aspects of synapse specific circuit assembly during development. While *Adgrl2* appears to be important for selective aspects of MEC circuitry in this study, future investigation into the additional genetic components that control *Adgrl2* independent mechanisms will be required.

## Data Availability

All data presented in this study are available upon request. Any additional information required to reanalyze the data reported in this paper is available upon request.

## References

[1] Wang Q, Gao E, Burkhalter A. Gateways of ventral and dorsal streams in mouse visual cortex. J Neurosci. 2011;31:1905–18. 21289200 10.1523/JNEUROSCI.3488-10.2011 PMC3040111

[2] Sur M, Rubenstein JLR. Patterning and plasticity of the cerebral cortex. Science. 2005;310:805–10. 16272112 10.1126/science.1112070

[3] Sur M, Leamey CA. Development and plasticity of cortical areas and networks. Nat Rev Neurosci. 2001;2:251–62. 11283748 10.1038/35067562

[4] Caballero‐Bleda M, Witter MP. Regional and laminar organization of projections from the presubiculum and parasubiculum to the entorhinal cortex: an anterograde tracing study in the rat. J Comp Neurol. 1993;328:115–29. 8429124 10.1002/cne.903280109

[5] Kononenko NL, Witter MP. Presubiculum layer III conveys retrosplenial input to the medial entorhinal cortex. Hippocampus. 2012;22:881–95. 21710546 10.1002/hipo.20949

[6] Nilssen ES, Doan TP, Nigro MJ, Ohara S, Witter MP. Neurons and networks in the entorhinal cortex: a reappraisal of the lateral and medial entorhinal subdivisions mediating parallel cortical pathways. Hippocampus. 2019;29:1238–54. 31408260 10.1002/hipo.23145

[7] Leamey CA, Merlin S, Lattouf P, Sawatari A, Zhou X, Demel N, et al. Ten_m3 regulates eye-specific patterning in the mammalian visual pathway and is required for binocular vision. Plos Biol. 2007;5:e241. 17803360 10.1371/journal.pbio.0050241 PMC1964777

[8] Pederick DT, Lui JH, Gingrich EC, Xu C, Wagner MJ, Liu Y, et al. Reciprocal repulsions instruct the precise assembly of parallel hippocampal networks. Science. 2021;372:1068–73. 34083484 10.1126/science.abg1774 PMC8830376

[9] Donohue JD, Amidon RF, Murphy TR, Wong AJ, Liu ED, Saab L, et al. Parahippocampal latrophilin-2 (ADGRL2) expression controls topographical presubiculum to entorhinal cortex circuit connectivity. Cell Reports. 2021;37:110031. 34818557 10.1016/j.celrep.2021.110031

[10] Toro DD, Carrasquero-Ordaz MA, Chu A, Ruff T, Shahin M, Jackson VA, et al. Structural basis of teneurin-latrophilin interaction in repulsive guidance of migrating neurons. Cell. 2020;180:323–339.e19. 31928845 10.1016/j.cell.2019.12.014 PMC6978801

[11] Sando R, Jiang X, Südhof TC. Latrophilin GPCRs direct synapse specificity by coincident binding of FLRTs and teneurins. Science (New York, NY). 2019;363:eaav7969. 10.1126/science.aav7969 10.1126/science.aav7969 PMC6636343 30792275

[12] Anderson GR, Maxeiner S, Sando R, Tsetsenis T, Malenka RC, Südhof TC. Postsynaptic adhesion GPCR latrophilin-2 mediates target recognition in entorhinal-hippocampal synapse assembly. J Cell Biol. 2017;216:jcb.201703042. 10.1083/jcb.201703042 PMC5674891 28972101

[13] Silva J-PP, Lelianova VG, Ermolyuk YS, Vysokov N, Hitchen PG, Berninghausen O, et al. Latrophilin 1 and its endogenous ligand Lasso/teneurin-2 form a high-affinity transsynaptic receptor pair with signaling capabilities. Proc Natl Acad Sci USA. 2011;108:12113–8. 21724987 10.1073/pnas.1019434108 PMC3141932

[14] Boucard AA, Maxeiner S, Südhof TC. Latrophilins function as heterophilic cell-adhesion molecules by binding to teneurins: regulation by alternative splicing. J Biol Chem. 2014;289:387–402. 24273166 10.1074/jbc.M113.504779 PMC3879561

[15] O’Sullivan, Wit ML, de J, Savas JN, Comoletti D, Otto-Hitt S, Yates JR, et al. FLRT proteins are endogenous latrophilin ligands and regulate excitatory synapse development. Neuron. 2012;73:903–10. 22405201 10.1016/j.neuron.2012.01.018 PMC3326387

[16] Jackson VA, Mehmood S, Chavent M, Roversi P, Carrasquero M, del Toro D, et al. Super-complexes of adhesion GPCRs and neural guidance receptors. Nat Commun. 2016;7:11184. 27091502 10.1038/ncomms11184 PMC4838878

[17] Lu YC, Nazarko OV, Sando R, Salzman GS, Li N-SS, Südhof TC, et al. Structural basis of Latrophilin-FLRT-UNC5 interaction in cell adhesion. Structure (London, England: 1993). 2015;23:1678–91. 26235030 10.1016/j.str.2015.06.024 PMC4851429

[18] Boucard AA, Ko J, Südhof TC. High affinity neurexin binding to cell adhesion G-protein-coupled receptor CIRL1/latrophilin-1 produces an intercellular adhesion complex. J Biol Chem. 2012;287:9399–413. 22262843 10.1074/jbc.M111.318659 PMC3308797

[19] Berns DS, DeNardo LA, Pederick DT, Luo L. Teneurin-3 controls topographic circuit assembly in the hippocampus. Nature. 2018;554:328. 29414938 10.1038/nature25463 PMC7282895

[20] Suh J, Rivest AJ, Nakashiba T, Tominaga T, Tonegawa S. Entorhinal cortex layer III input to the hippocampus is crucial for temporal association memory. Science. 2011;334:1415–20. 22052975 10.1126/science.1210125

[21] Madisen L, Zwingman TA, Sunkin SM, Oh SW, Zariwala HA, Gu H, et al. A robust and high-throughput Cre reporting and characterization system for the whole mouse brain. Nat Neurosci. 2010;13:133–40. 20023653 10.1038/nn.2467 PMC2840225

[22] Friard O, Gamba M. BORIS: a free, versatile open‐source event‐logging software for video/audio coding and live observations. Methods Ecol Evol. 2016;7:1325–30.

[23] Barnes CA. Memory deficits associated with senescence: a neurophysiological and behavioral study in the rat. J Comp Physiol Psych. 1979;93:74–104. 10.1037/h0077579 221551

[24] Kawano J, Fujinaga R, Yamamoto-Hanada K, Oka Y, Tanizawa Y, Shinoda K. Wolfram syndrome 1 (Wfs1) mRNA expression in the normal mouse brain during postnatal development. Neurosci Res. 2009;64:213–30. 19428703 10.1016/j.neures.2009.03.005

[25] Groen T, van, Miettinen P, Kadish I. The entorhinal cortex of the mouse: organization of the projection to the hippocampal formation. Hippocampus. 2003;13:133–49. 12625464 10.1002/hipo.10037

[26] Fuchs EC, Neitz A, Pinna R, Melzer S, Caputi A, Monyer H. Local and distant input controlling excitation in layer II of the medial entorhinal cortex. Neuron. 2016;89:194–208. 26711115 10.1016/j.neuron.2015.11.029 PMC4712190

[27] Carola V, D’Olimpio F, Brunamonti E, Mangia F, Renzi P. Evaluation of the elevated plus-maze and open-field tests for the assessment of anxiety-related behaviour in inbred mice. Behav Brain Res. 2002;134:49–57. 12191791 10.1016/s0166-4328(01)00452-1

[28] Wilson DIG, Langston RF, Schlesiger MI, Wagner M, Watanabe S, Ainge JA. Lateral entorhinal cortex is critical for novel object‐context recognition. Hippocampus. 2013;23:352–66. 23389958 10.1002/hipo.22095 PMC3648979

[29] Dees RL, Kesner RP. The role of the dorsal dentate gyrus in object and object-context recognition. Neurobiol Learn Mem. 2013;106:112–7. 23880567 10.1016/j.nlm.2013.07.013

[30] Beer Z, Vavra P, Atucha E, Rentzing K, Heinze H-J, Sauvage MM. The memory for time and space differentially engages the proximal and distal parts of the hippocampal subfields CA1 and CA3. Plos Biol. 2018;16:e2006100. 30153249 10.1371/journal.pbio.2006100 PMC6136809

[31] Montchal ME, Reagh ZM, Yassa MA. Precise temporal memories are supported by the lateral entorhinal cortex in humans. Nat Neurosci. 2019;22:284–8. 30643291 10.1038/s41593-018-0303-1 PMC6592045

[32] Wang Q, Marcucci F, Cerullo I, Mason C. Ipsilateral and contralateral retinal ganglion cells express distinct genes during decussation at the optic chiasm. eNeuro. 2016;3:ENEURO.0169-16.2016. 27957530 10.1523/ENEURO.0169-16.2016 PMC5136615

[33] Masuda A, Nishida K, Ajima R, Saga Y, Bakhtan M, Klar A, et al. A global gene regulatory program and its region-specific regulator partition neurons into commissural and ipsilateral projection types. Sci Adv. 2024;10:eadk2149. 38781326 10.1126/sciadv.adk2149 PMC11114196

[34] Li J, Shalev-Benami M, Sando R, Jiang X, Kibrom A, Wang J, et al. Structural basis for teneurin function in circuit-wiring: a toxin motif at the synapse. Cell. 2018;173:735–748.e15. 29677516 10.1016/j.cell.2018.03.036 PMC5912346

[35] Jackson VA, del Toro D, Carrasquero M, Roversi P, Harlos K, Klein R, et al. Structural basis of latrophilin-FLRT interaction. Structure. 2015;23:774–81. 25728924 10.1016/j.str.2015.01.013 PMC4396693

[36] Li J, Xie Y, Cornelius S, Jiang X, Sando R, Kordon SP, et al. Alternative splicing controls teneurin-latrophilin interaction and synapse specificity by a shape-shifting mechanism. Nat Commun. 2020;11:2140. 32358586 10.1038/s41467-020-16029-7 PMC7195488

[37] Zhang X, Lin P-Y, Liakath-Ali K, Südhof TC. Teneurins assemble into presynaptic nanoclusters that promote synapse formation via postsynaptic non-teneurin ligands. Nat Commun. 2022;13:2297. 35484136 10.1038/s41467-022-29751-1 PMC9050732

[38] Sando R, Südhof TC. Latrophilin GPCR signaling mediates synapse formation. Elife. 2021;10:e65717. 33646123 10.7554/eLife.65717 PMC7954527

[39] Pederick DT, Perry-Hauser NA, Meng H, He Z, Javitch JA, Luo L. Context-dependent requirement of G protein coupling for Latrophilin-2 in target selection of hippocampal axons. eLife. 2023;12:e83529. 36939320 10.7554/eLife.83529 PMC10118387

[40] Donato F, Jacobsen RI, Moser M-B, Moser EI. Stellate cells drive maturation of the entorhinal-hippocampal circuit. Science. 2017;355:eaai8178. 28154241 10.1126/science.aai8178

[41] Seng C, Luo W, Földy C. Circuit formation in the adult brain. Eur J Neurosci. 2022;56:4187–213. 35724981 10.1111/ejn.15742 PMC9546018

